# The Effect of Targeted Memory Reactivation on Dogs’ Visuospatial Memory

**DOI:** 10.1523/ENEURO.0304-20.2024

**Published:** 2025-02-11

**Authors:** Henrietta Bolló, Cecília Carreiro, Ivaylo Borislavov Iotchev, Ferenc Gombos, Márta Gácsi, József Topál, Anna Kis

**Affiliations:** ^1^Institute of Cognitive Neuroscience and Psychology, Research Centre for Natural Sciences, Budapest 1117, Hungary; ^2^Department of Ethology, ELTE Eötvös Loránd University, Budapest 1117, Hungary; ^3^Laboratory for Psychological Research, Pázmány Péter Catholic University, Budapest 1088, Hungary; ^4^HUN-REN-ELTE-PPKE Adolescent Development Research Group Budapest, Budapest 1075, Hungary; ^5^HUN-REN-ELTE Comparative Ethology Research Group, Budapest 1117, Hungary

**Keywords:** *Canis familiaris*, sleep, targeted memory reactivation, translational modeling, visuospatial memory

## Abstract

The role of sleep in memory consolidation is a widely discussed but still debated area of research. In light of the fact that memory consolidation during sleep is an evolutionary adaptive function, investigating the same phenomenon in nonhuman model species is highly relevant for its understanding. One such species, which has acquired human-analog sociocognitive skills through convergent evolution, is the domestic dog. Family dogs have surfaced as an outstanding animal model in sleep research, and their learning skills (in a social context) are subject to sleep-dependent memory consolidation. These results, however, are correlational, and the next challenge is to establish causality. In the present study, we aimed to adapt a TMR (targeted memory reactivation) paradigm in dogs and investigate its effect on sleep parameters. Dogs (*N* = 16) learned new commands associated with different locations and afterward took part in a sleep polysomnography recording when they were re-exposed to one of the previously learned commands. The results did not indicate a cueing benefit on choice performance. However, there was evidence for a decrease in choice latency after sleep, while the density (occurrence/minute) of fast sleep spindles was also notably higher during TMR recordings than adaptation recordings from the same animals and even compared with a larger reference sample from a previous work. Our study provides empirical evidence that TMR is feasible with family dogs, even during a daytime nap. Furthermore, the present study highlights several methodological and conceptual challenges for future research.

## Significance Statement

A targeted memory reactivation (TMR) paradigm was adapted to family dogs for the first time. The goal of TMR is to enhance memory consolidation during sleep by replaying some of the previously learned cues in non-REM. There is evidence of TMR effect in humans and rodents. Here we aimed to adapt a TMR protocol to family dogs with a noninvasive polysomnography technique. Dogs were learning new commands associated with different locations, and during sleep they heard one of the new commands. After sleep, dogs were asked to answer to the new commands by going to the previously learned places. Although their accuracy did not increase after TMR sleep, there was a slight decrease in response latency.

## Introduction

The effect of sleep on memory consolidation is widely discussed (for a review, see Diekelmann and Born, 2010), and many unresolved debates still exist about the specific underlying mechanisms (Genzel et al., 2014) by which memories are formed or strengthened during sleep. One area of halted progress concerning sleep physiology and learning is the issue of cross-species comparability, in which human–rodent comparisons predominate (Hars and Hennevin, 1987; Mölle et al., 2009; Barnes and Wilson, 2014; Latchoumane et al., 2017).

There is a growing body of evidence that dogs (*Canis familiaris*) can be a unique translational model for human sleep physiology (Kis et al., 2014; Bunford et al., 2017; Bódizs et al., 2020; Iotchev and Kubinyi, 2021). It has recently been demonstrated that dogs’ learning skills, in a social context, are linked to sleep-dependent memory consolidation, as indicated by EEG spectral changes (Kis et al., 2017) and sleep spindles (Iotchev et al., 2017, 2020b). Most of these findings are correlational, however, and the next challenge is to establish or approximate causality in this model species for which noninvasive research will likely become the new standard in the near future (Bailey and Pereira, 2018).

Based on human research, targeted memory reactivation (TMR), a tool that enhances memory consolidation during sleep via repeatedly presenting a cue previously associated with a target, appears promising toward testing for an active role of sleep in memory consolidation (Schouten et al., 2017). In the present study, we aim to adapt the TMR paradigm in family dogs (dogs who live in households with their owners) and investigate its effect on their sleep parameters and learning performance.

In a typical TMR paradigm, subjects are exposed to different sensory (most commonly auditory) cues while being trained on a memory task. During subsequent sleep, the cues are re-presented without waking the subject. Afterward, cued tasks are more easily recalled compared with those without cueing. A commonly used subtype of targeted memory recall is the visuospatial TMR task, during which participants are asked to associate different object locations with congruent sound cues (e.g., picture of a cat with “meow” sound and picture of a dog with “bark” sound). Learning is followed by a sleep session, during which half of the cue sounds (e.g., bark or meow) are presented during non-REM sleep. At the subsequent postsleep recall in the picture location task, subjects’ accuracy for locations associated with cued sounds are increased compared with the uncued ones (Rudoy et al., 2009). Moreover, this benefit can also be detected without any apparent semantic relationship between the sounds and pictures (e.g., “meow” paired with Brad Pitt; Antony et al., 2018). Furthermore, the efficiency of cueing depends on presleep performance (Creery et al., 2015); those participants who showed higher accuracy in the presleep task benefited more from cueing.

Although TMR in humans is a useful tool to accelerate memory consolidation and has several potential clinical applications (Schouten et al., 2017), the underlying mechanisms are still barely unexplored. According to the “active system hypothesis,” memory consolidation is based on the active induction of memory reactivations (Born et al., 2006; Diekelmann and Born, 2010; Rasch and Born, 2013). Reactivations during non-REM slow wave sleep induces a system-level communication between hippocampal networks and the neocortex. Furthermore, sleep duration (Creery et al., 2015) and delta power (Oudiette et al., 2013; Creery et al., 2015), changes in negative-to-positive slow oscillation slope (Rihm et al., 2014), theta power (Schreiner et al., 2015), as well as spindle power and density (Rihm et al., 2014; Creery et al., 2015) were positively correlated with cueing benefits of TMR, but other studies failed to confirm these associations (Diekelmann et al., 2012; Cox et al., 2014). Although Cox et al. (2014) did not find a direct link between memory performance and sleep parameters, they detected a modulatory effect in spindle activity and density in response to the cueing.

In light of the fact that memory consolidation during sleep is an evolutionary adaptive function (Vorster and Born, 2015), investigating the same phenomenon in nonhuman model species is highly relevant for its understanding. Memory consolidation has been extensively studied in rodents; however, TMR protocols differ greatly from those used in human studies and are focused solely on fear conditioning during sleep (Hars and Hennevin, 1987; Barnes and Wilson, 2014). In order to maximize ecological validity in such comparative studies, it is reasonable to choose a model species, which evolutionarily adapted to the anthropogenic environment: dogs. Due to their long domestication history (∼18,000–32,000 years; Druzhkova et al., 2013), dogs’ sociocognitive skills adapted to the same environmental challenges as humans’ (Hare and Tomasello, 2005; Topál et al., 2009; Parker et al., 2010). Using dogs as model animals in neurocognitive research has several advantages considering that (1) dogs are willing to cooperate during the measurement at least to the same extent as children, (2) a relatively large number of subjects are available, (3) subjects have more diverse genotypes and phenotypes than mice or rats, and (4) measurements can be performed in the subjects’ natural environment similarly to the methods used in children (Kis et al., 2014; Bunford et al., 2017; Bódizs et al., 2020).

A noninvasive polysomnography technique has already been adapted for dogs (Kis et al., 2014), allowing for the design of neurocognitive studies that are directly comparable with those in humans. In their study, Kis et al. (2017) showed the first evidence that dogs’ learning skills are related to sleep-dependent memory consolidation. Dogs’ performance in a command learning task improved after a 3-h-long sleep compared with the presleep baseline, and this improvement correlated with different aspects of EEG spectral power (REM sleep delta and beta activity). Reanalyzing the same dataset looking for sleep spindles (using an automated algorithm) revealed that the number of spindles/minute was larger in the learning condition (compared with control) and correlated with the learning gain during sleep (Iotchev et al., 2017, 2020a).

Here, we aim to investigate the potential effect of TMR on pet dogs’ memory performance in a visuospatial task using the abovementioned noninvasive EEG method. We hypothesize that, similarly to humans (Creery et al., 2015), dogs’ postsleep performance would specifically increase for cued (as opposed to noncued) commands, and this cueing benefit is expected to be higher for previously more successful dogs. Furthermore, the effect of cueing on sleep parameters, including spectral data and spindle density, is investigated (Oudiette et al., 2013; Rihm et al., 2014; Creery et al., 2015).

## Materials and Methods

### Subjects

A total of 26 task-naive adult pet dogs and their owners were recruited on a voluntary basis from the subject pool of the Family Dog Project (Abdai and Miklósi, 2015). This sample size is slightly larger than originally planned in the preregistered manuscript (*N* = 20) due to a higher-than-expected dropout rate. The sole requirement for participation was that dogs should be at least 1 year old. Participating in the sleep EEG research generally does not require prior training. Regarding sleep quality, a minimum of one sleep cycle at the second TMR occasion is needed to include the dog in the final sample. Another inclusion criterion to participate at the second occasion of the test was defined based on side bias during the familiarization phase. Of the recruited participants, *N* = 2 dogs showed signs of frustration during the adaptation sleep leading to the termination of their measurements; *N* = 2 dogs showed center bias (they always chose the center container) during the baseline trials; *N* = 2 did not return for the second occasion, and data recordings were unsuccessful for four dogs due to technical issues with the video recordings or the polysomnography data registration. The final sample consisted of 16 dogs who fully completed the protocol: nine males; *M*(SD) age, 4.49(2.59) years from 5 breeds (Borzoi, Dachshund, Labrador Retriever, Dobermann, and a Bichon Havanese) and 11 mixed breed dogs.

### General procedure

Dogs came to the laboratory on two occasions with ∼1 week interval in between. The first occasion was aimed to familiarize the subjects with the laboratory, the experimenter, the containers, and the electrode placement to avoid a phenomenon known in the literature as the first-night effect (Reicher et al., 2020). The second occasion was the TMR session when dogs first participated in a visuospatial learning task, where they learned to respond to three verbal commands, and then in a sleep polysomnography recording, when they were exposed to one of the previously learned commands. After sleep, dogs again completed the visuospatial task.

**Figure 1.**
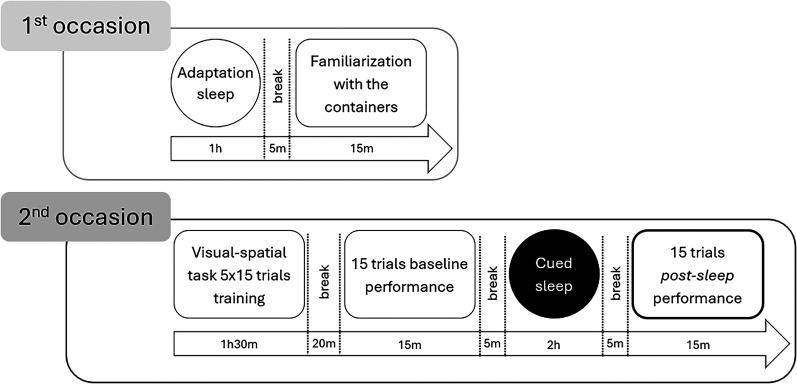
Timeline of the general procedure.

We previously estimated (based on Gergely et al., 2014) that 75 trials would be needed in order to avoid ceiling effects but have all dogs learn the task reliably. Training was carried out in 15-trial blocks. After each block, there was a short 5 min break, and, after the training trials, there was a longer 20 min break, during which dogs could take a walk before the baseline test trials. After the break, 15 test trials were carried out to set the baseline. This was followed by the 2-h-long polysomnography measurement and the subsequent 15 test trials. Figure 1 presents the timeline of the general procedure.

### Visuospatial task

#### Familiarization (first occasion)

**Figure 2.**
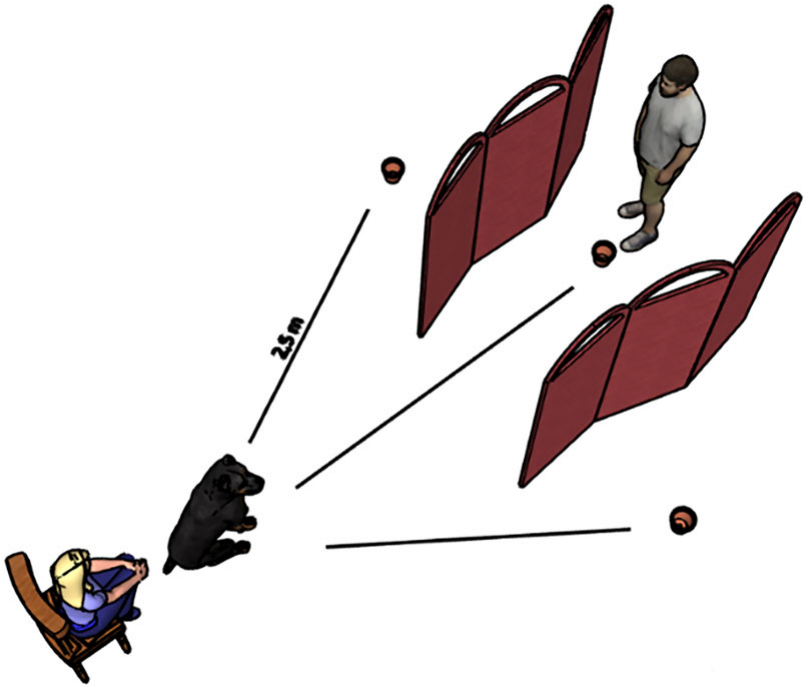
Experimental layout of the visuospatial task.

In order to make sure that the dog knew that there was a food bait in the container, a short familiarization phase was carried out on the first occasion. Following the adaptation sleep, dogs were led to the behavior laboratory and allowed a free exploration of ∼5 min, after which the owner was asked to call the dog and hold it next to them. The experimenter, standing at a 1 m distance, baited one container (visible to the dog) and then put the container on the floor. After this, the dog was released and allowed to move *ad libitum* and eat the bait. As a next step, the experimenter put three containers in a semicircular arrangement (Fig. 2). The containers were placed 2.5 m away from the dog, and each container was separated by a screen to prevent the dog from eating all the food baits at once and to familiarize the dog with the screens. All three containers were baited (with the experimenter turning her back to the subject so the dog could not see the baiting), and the containers were placed on the floor in a left-to-right sequence relative to the perspective of the experimenter facing the dog. When the experimenter returned to the center, she nodded to the owner to release the dog while looking down to avoid eye contact with the dog. The dog was allowed to find all three food baits. On the bottom of each container, we attached a food bait with a tape to prevent odor cues from helping the dog identify the correct container during all trials (familiarization, training, test).[Fig eN-NWRGR-0304-20F3][Fig eN-NWRGR-0304-20F4]

**Figure 3. eN-NWRGR-0304-20F3:**
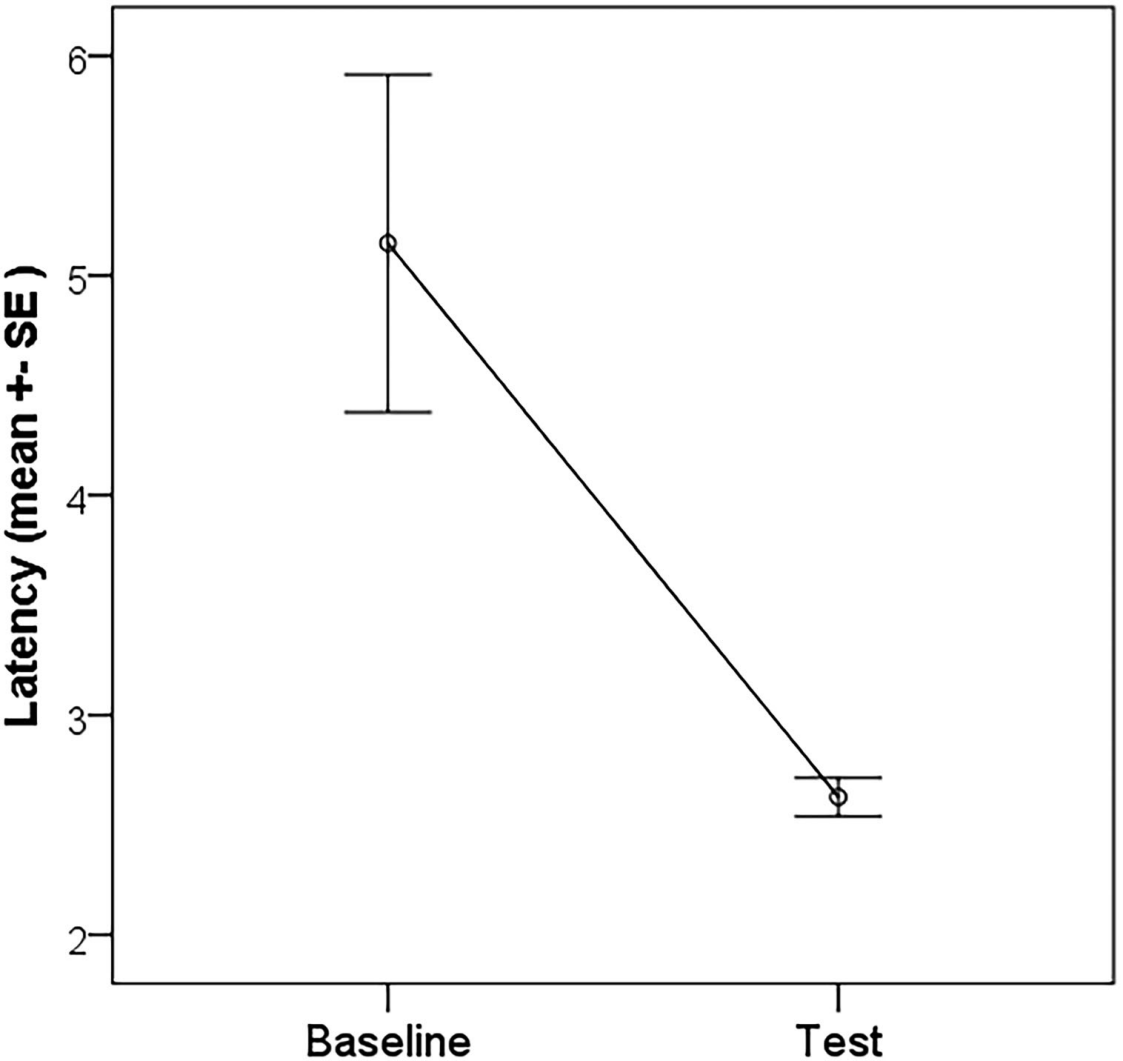
Decrease in choice latency between the baseline and test sessions (main effect).

**Figure 4. eN-NWRGR-0304-20F4:**
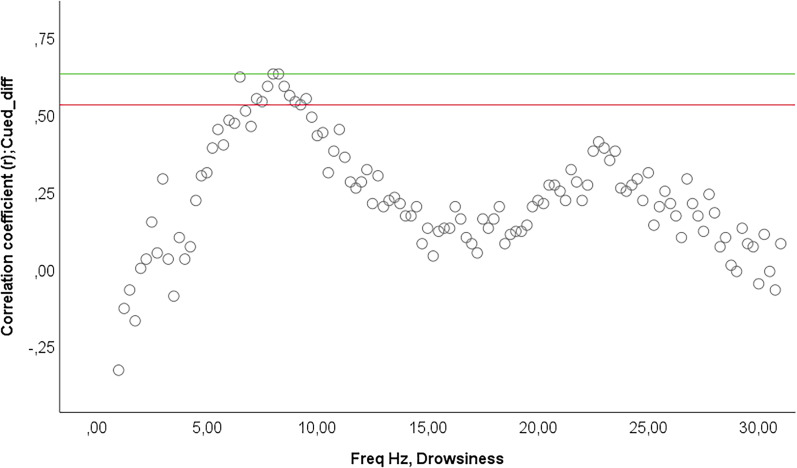
Correlation between the difference accuracy scores of the trials when the cued nonword was presented and drowsiness EEG power spectrum. Correlation coefficients for the Fz EEG channel is shown with points below the green line (*r* = 0.63) and above the red line (*r* = 0.53) indicating significant (*p* < 0.05) associations for the given frequency bin.

#### Training phase (second occasion)

The aim of the training was for dogs to associate three nonwords (used in a command-like context) with one of three hiding locations. The specific locations (right, center left) signaled by the different nonwords (“gyem,” “szan,” “lok”) were counterbalanced across subjects. The order in which the different containers were baited followed a fixed semirandom order (ABC, ACB, CAB, CBA, BAC) in a way that during the 15-trial block, all three containers were baited exactly five times, and the same container was never baited twice in a row. As food bait, we used dry cat food because it is small and dogs like it. For smaller dogs (<6 kg), the food bait was split to avoid big motivational differences. Baiting was invisible for both the dog and the owner as the experimenter turned her back toward them during baiting.

For the first block of 15 trials after baiting one of the containers, the experimenter placed down all three containers at their predetermined positions. Then the experimenter stood 50 cm behind the center container, faced the dog, and called its name followed by the corresponding nonword. Because of this setup, it is important to note that the cued location was either the left or the right side for all dogs, but never the center, since dogs tend to make center choices in situations when they are uncertain of the correct response (Range et al., 2011). On the other hand, we used three locations because dogs have a tendency to learn easily by exclusion (Kaminski et al., 2004); therefore using only two containers would increase the probability of applying this technique and allow dogs to learn only one command, and all other cues would be associated with the other side.

During the training, after calling the dog and giving the command, if the experimenter saw that the dog was hesitating or did leave the owner's site, she repeated the command up to a maximum of three times. After a 2 s delay, the experimenter pointed to the baited container making a step toward the location. The dog was released, while the experimenter kept pointing to the correct location to ensure the highest possible success rate at this phase of learning.

For the following blocks of trials, the experimenter decreased the saliency of pointing step by step, e.g., starting from the second block, only the pointing was presented without the body movement, and, by the end of the third block, no pointing was presented for the fastest learning dogs. During training, the experimenter fine-tuned the characteristics of the accompanying cues for each dog in order to maximize learning. For example, the second block of trials mostly started without the experimenter's body movement, but if a subject made consecutive wrong choices, the previously used aiding cue was used again. The same rule was applied to switch from arm-pointing to no pointing and generally throughout the training session (75 trials). During the test session, however, the protocol was standardized for all subjects independently of individual performance.

#### Test phases (second occasion)

After the training and a longer break (20 min), the first block of test trials, the baseline was carried out. The second 15-trial block served to measure the dogs’ postsleep performance. In both tests, the location of the baited container followed the same fixed semirandom order as in the training trials (in both 15-trial blocks, each location was baited five times). During the test phases, the owner wore a headset and listened to music to avoid pressing the dog to the correct direction.

Standing on the same spot as during the training trials, the experimenter called the dog's name and uttered the same commands as during the training without pointing or (any other movement) and looked down to avoid eye contact with the dog. The owner released the dog that could choose one container and then the experimenter picked up the other containers.

### Polysomnography recordings

The recordings were always scheduled for afternoon between 13:00 and 17:00 because apart from night time, dogs, similar to humans, show the highest propensity to sleep during the afternoon (Takahashi et al., 1972). We followed a validated canine polysomnography protocol (Reicher et al., 2020).

After a few minutes of *ad libitum* exploration in the sleeping laboratory (a room adjacent to the behavioral laboratory), the owner settled down on the mattress together with the dog. During electrode placement, all dogs were reinforced using social reinforcement (e.g., petting, praise) and/or food reward. Two electrodes were placed on the right and left zygomatic arch next to the eyes (F7, F8) and two other ones over the anteroposterior midline of the skull (Fz, Cz). All four EEG electrodes were referred to the G2 electrode which was in the posterior midline of the skull (occiput; external occipital protuberance). The ground electrode (G1) was attached on the left *musculus temporalis*. Electrodes were placed bilaterally on the *musculus iliocostalis dorsi* for electromyography and over the second rib for electrocardiography (ECG). Respiratory movements were also monitored by a respiratory belt attached to the chest. For the recordings, gold-coated Ag/AgCl electrodes were used, secured by Signaspray Electrode Solution (Parker Laboratories) and EC2 Grass Electrode Cream (Grass Technologies). The impedance values of the EEG electrodes were kept under 20 kΩ during the recordings. The signals were collected, prefiltered, amplified, and digitized at a sampling rate of 1,024 Hz per channel, using the 25-channel SAM 25R EEG System (Micromed) and the System Plus Evolution software with second-order filters at 0.016 Hz (high-pass) and 70 Hz (low-pass).

### TMR paradigm

After placing the electrodes, the experimenter left the owner and the dog in the sleeping room and monitored the recording from the adjacent room. During the adaptation sleep (first occasion), no cues were given during the measurement. Based on the results from a recent study (Bolló et al., 2020) on valid differentiation of non-REM and REM phases of the sleeping dog via real-time online monitoring, during the TMR (second occasion), the nonword corresponding to the left or right location (counterbalanced across subjects) was repeatedly played back when the dog was in non-REM sleep. The command was played back when the subject was deemed to be in non-REM sleep for approximately two 30 s epochs (Rudoy et al., 2009; Creery et al., 2015), and the sound playback was manually interrupted by the experimenter if any sign of awakening, arousal, or REM sleep was detected (Schreiner et al., 2015). The chosen cue (nonword corresponding to left/right) was played every 5 s, yielding a stimulation of ∼5 min.

### Sleep spindle detection

Sleep spindles were automatically detected in line with the method introduced in Iotchev et al. (2017) in turn based on the work of Nonclercq et al. (2013). The algorithm uses an amplitude–frequency criterion for initial detections, which are then used to readjust the criterion by calculating maximum likelihood estimates for the true means and standard deviations of spindle amplitude and frequency. The search is restricted to non-REM segments of the signal and 0.5-s-long time-windows (minimum duration of a spindle). Detections were divided into slow and fast using the common 13 Hz threshold (fast spindles defined as ≥13 Hz).

### Variables and statistical analysis

Polysomnography recordings of dog sleep were coded with a self-developed software (Fercio) according to standard criteria for dogs (Kis et al., 2014). Wakefulness, drowsiness, non-REM, and REM sleep were coded in 20 s epochs by inspecting the EEG, EOG, and ECG channels. Compared with human sleep scoring, a notable difference in canine sleep scoring arises from the characteristics of drowsiness. Drowsiness is characteristic of insectivore and carnivore mammals because in these taxa the transition from wakefulness to sleep is not as clear as in humans; therefore it bears characteristics of both human Stage 1 non-REM sleep and quiet awake (Zepelin, 1994). Due to this difference, we used two approaches to determine wakefulness after sleep onset (WASO) and sleep latency. The following nine variables were exported from the hypnograms: sleep efficiency (time spent asleep relative to the total length of the recording, %), relative wake duration (time spent awake relative to the total length of the recording, %), WASO1 (waking after sleep onset after first drowsiness, min), WASO2 (waking after sleep onset after first non-REM sleep, min), Sleep Latency 1 (until first drowsiness, min), Sleep Latency 2 (until first non-REM sleep, min), relative drowsiness duration (%), relative non-REM duration (%), and relative REM duration (%). Artifact rejection was carried out manually on 4 s epochs before further automatic analyses on all recordings. Average power spectral densities (1–30 Hz) were calculated by a fast Fourier transformation algorithm, applied to the 50% overlapping, Hanning-tapered 4 s windows of the EEG signal of the Fz–Cz derivation. Power spectra were calculated separately for drowsiness and non-REM and REM sleep on 0.25 Hz frequency bins. No spectral analysis was carried out for the EEG signal of the wake stage, due to the high proportion of artifacts (resulting from muscle tone). Relative EEG power was calculated for each frequency bin as the percentage of total power (e.g., the absolute power value of the given bin divided by the sum of absolute power values for the 1–30 Hz frequency range). We calculated relative spectrum power in the four frequency ranges of delta (1–4 Hz), theta (4–8 Hz), alpha (8–12 Hz), and beta (12–30 Hz). Additionally, a bin-by-bin analysis was carried out on the full (1–30 Hz) spectrum with 0.25 Hz bins. In order to address the issue of multiple comparisons, a Rüger correction was used on the bin-by-bin results (Abt, 1987). Rüger's areas are defined as sets of conventionally significant (*p* < 0.05) results, which are accepted or rejected as significant as a whole, rather than based on individual results of statistical tests. Taking the results of the statistical tests as a matrix, Rüger's areas can be defined along the dimension of frequency bins. Starting from the lower frequencies, a Rüger's area is the range of all the neighboring, consecutive frequency bins, which contain a significant result surrounded by bins containing nonsignificant results. After defining these areas of significance, the number of significant results within the area was calculated, and we could investigate whether at least half of these results are significant at least at half of the conventional *p* = 0.05 significance level (i.e., whether they were below 0.025), and at least one-third of them are significant at least at a third of the conventional *p* = 0.05 significance level (below 0.0167). If both of these conditions are fulfilled, the area as a whole was considered as significant.

Because the literature on TMR and sleep spindles suggests an increase in sleep spindle occurrence during TMR cueing (Cairney et al., 2018; Wang et al., 2019; Carbone et al., 2021; Hopper, 2021), the analysis for possible effects of TMR on sleep spindles was based on a comparison between TMR and non-TMR recordings. The latter included adaptation recordings of the same dogs (*N* = 16) but also a larger reference population taken from Iotchev et al. (2019), comprising *N* = 146 valid Fz recordings and *N* = 85 valid Cz recordings. The larger reference population was included because of the small sample size within the TMR experiment and adaptation recordings not being an optimal control condition. We compensated for the latter with a more representative estimate of “normal” sleep spindle density (occurrence/minute) provided by the larger sample size. A GLMM was used to account for values coming from the same subjects. The analysis was conducted separately for slow and fast sleep spindles, since previous studies reported subtype-specific increases during TMR (Creery et al., 2015; Cairney et al., 2018; Carbone et al., 2021). We focused only on sleep spindle density, since of all spindle features studied so far, it is the most consistent predictor of cognition in dogs (Iotchev et al., 2017, 2020a, 2024). Raw data is available at https://osf.io/96hj7/.

Video recordings of the visuospatial task were coded by a blind rater for the given nonword, using Solomon Coder. Two variables were coded in both the baseline and the postsleep trials: accuracy (0, if the dog does not choose the baited container; 1, if the dog chooses the baited container) and latency (elapsed time between the owner's release and the dog’ nose reaching the container). Dogs who performed out of the 10th or 90th percentile from the mean of accuracy were identified as outliers.

Head orientation was coded during the baseline trials and the test trials. During the baseline trials, dogs looked toward the containers for 97% of the total trial time and during the test trials they looked toward the containers for 79% of the total trial time, which confirmed that dogs were paying attention during the task. Furthermore, during the training phase, the following variables were coded: help (1, pointing with a large arm movement accompanied by whole-body movements; 2, pointing with a small arm movement while the body remains still; 3, no pointing) and also an accuracy (0, if the dog does not choose the baited container; 1, if the dog chooses the baited container, and side score (1, left container; 2, center container; 3, right container) to test the efficacy of the training and the dogs’ potential preference.

The effect of TMR on dogs’ performance was analyzed by a generalized linear mixed-effect model (GLMM) using the binomial distribution. The outcome variable was the accuracy (0, inaccurate; 1, accurate) of the 30 trials (15 baseline + 15 test trials), and the model included test time (baseline or test) and container + side combined (1, cued left; 2, cued right; 3, uncued left; 4, uncued right; 5, center) and the trial number (from 1 to 15) as fixed factors and ID of the dog as random factors. The same fixed and random factors were included in the analysis of latency scores with a GLMM.

Furthermore, a *Cueing benefit* was calculated as postsleep accuracy − baseline accuracy of cued trials and included in a GLMM as a target variable, while the abovementioned same fixed and random factors were also included to see if cueing benefit is higher for previously more successful dogs (Creery et al., 2015). Furthermore, sleep duration (Creery et al., 2015), delta power (Oudiette et al., 2013; Creery et al., 2015), and theta power (Schreiner et al., 2015) were expected to positively correlate with *Cueing benefit*, and we tested the correlations with all macrostructural variables and bin-by-bin analysis on the full (1–30 Hz) spectrum with 0.25 Hz resolution. Furthermore, we planned to include cueing benefit in a GLMM as a target variable while including all four frequency ranges of delta (1–4 Hz), theta (4–8 Hz), alpha (8–12 Hz), and beta (12–30 Hz) as covariates because this was the first time testing a TMR protocol among dogs using noninvasive polysomnography; therefore, it was rather explorative regarding these variables.

## Results

Table 1 presents the descriptive statistics of dogs’ sleep macrostructure data. A visual examination of the current dataset reveals a sleep macrostructure similar to that reported in an earlier polysomnography validation study (Kis et al., 2014), with overlapping mean ± SD values for all variables and similarly large individual variation. This suggests that the auditory cueing during sleep did not substantially affect sleep quality.

**Table 1.** Descriptive statistics of the macrostuctural data of the dogs included in the present study (*N* = 16) contrasted to macrostructural data from Kis et al. (2014)

	Mean ± SD		Min–max	
	TMR	Kis et al.	TMR	Kis et al.
Sleep efficiency (%)	78.9 ± 20.4	42.7 ± 23.3	15.9–96.44	7.7–88.9
Sleep latency (min)	8.44 ± 3.9	28.3 ± 29.6	1.00–9.67	4.3–136.0
Drowsiness (min)	24.6 ± 14.7	37.6 ± 20.1	7.3–51.33	3.0–91.0
Non-REM (min)	58.5 ± 21.6	21.6 ± 21.5	11.7–96.00	0.0–89.3
REM (min)	12.4 ± 7.8	16.5 ± 20.5	0.00–27.67	0.0–60.3

The GLMM on *Accuracy scores* showed no significant main effect or interactions of trial number (1–30), test time (baseline or test) and container + side combined factors (all *p* > 0.05). However, the GLMM model on *Latency scores* showed a significant main effect of test time (*F*_(1,373) _= 11.98; *p* < 0.01) indicating shorter choice latencies after sleep [*M*(SD)_baseline _= 5.25(4.39) s; *M*(SD)test = 4.22(3.42) s]. There were no other significant main effects or interactions (*p* > 0.05; Fig. 1). The analysis of the *Cueing benefit* did not show any significant main effects or interaction of *Test time* and *Container + side combined*, and the presleep performance of the dogs also had no effect (all *p* > 0.05; see Figs. 3, 4).

Regarding the sleep macrostuctural variables, neither *Accuracy scores* nor *Cueing benefit* was related to either of them (all *p* > 0.05). The bin-by-bin analysis of the EEG spectrum in the different sleep stages revealed a significant Rüger area from 7 to 9.5 Hz (alpha range) with positive *r* values during drowsiness on the Fz channel and the *Accuracy scores* of the cued trials (presleep). No other correlations were found between sleep EEG spectrum and *Cueing benefit*.

**Figure 5.**
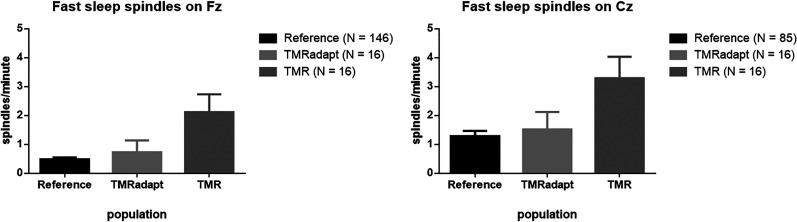
Fast spindle density for TMR and non-TMR recordings.

Next sleep spindle density (occurrence/minute) was compared between TMR and non-TMR recordings. For fast sleep spindles on Fz, there was a main effect of population (*F*_(2,175) _= 15.151; *p* < 0.001). Fast spindle density on Fz from TMR recordings was significantly higher than in the reference population of 146 adaptation recordings (TMR vs reference *M* ± SE, 2.11 ± 0.3 vs 0.48 ± 0.1; *p* < 0.001) and also higher than in adaptation recordings from the same dogs (TMR vs TMR adaptation *M* ± SE, 2.11 ± 0.3 vs 0.73 ± 0.3; *p* = 0.001). Fast spindle density was not different between adaptation recordings from the TMR experiment and the reference population (*p* = 0.410). Fast spindle density also differed between populations on Cz (*F*_(2,114) _= 6.900; *p* = 0.001). The highest fast spindle density was observed for TMR recordings (TMR vs reference *M* ± SE, 3.29 ± 0.5 vs 1.29 ± 0.2; *p* < 0.001; TMR vs TMR adaptation *M* ± SE, 3.29 ± 0.5 vs 1.52 ± 0.5; *p* = 0.013). There was no difference between adaptation recordings from the TMR experiment and the reference population on Cz (*p* = 0.667). Differences in slow spindle density did not reach significance on Fz (*p* = 0.055) nor on Cz (*p* = 0.439). The results are summarized in Figure 5.

### Explorative analyses and results

The observation that fast sleep spindles were more abundant in TMR than non-TMR recordings and that response latency was reduced postsleep prompted us to further explore both variables and their potential to uncover more subtle indications of TMR-induced learning. The rationale for these additional analyses and their results are detailed below.

#### Latency reduction and its derivatives as a measure of learning

The overall low accuracy/success rate in the here-reported learning paradigm may suggest that no learning took place, but the overall reduction in response latency, which was also observed in human sleep-dependent motor learning (Tamaki et al., 2008), warranted a closer look at the dynamics of latency reduction. Latency reduction was separately calculated for correct and incorrect trials by calculating the ratio latency presleep/latency postsleep, wherein a higher number express a larger reduction in response latency from pre- to postsleep testing. There was no significant difference in latency reduction between correct and incorrect trials (*p* = 0.352), but notably for 68.8% of the sample (11 of 16 dogs), the latency reduction was bigger for correct trials. A finer distinction by looking separately into cued, uncued, and center trials resulted in even smaller subsamples with even more limited implications (too low statistical power) and were therefore not further explored. We calculated a measure of relative latency reduction as the ratio latency reduction for correct trials divided by the latency reduction for incorrect trials. It is used below for analyzing the possible effect of fast sleep spindles on sleep-dependent learning. We reasoned that a stronger latency reduction for the correct responses may better indicate ongoing learning, since choice behavior in dogs can in theory be compromised by several factors. These include the common for dogs’ side bias (Gácsi et al., 2009; Bolló et al., 2023), a preference to consistently pick one side in two-way choice tasks. Related to the side bias and possibly adding to its effect is a perseverance to the first choice–outcomes, which Gácsi et al. (2009) described in a subset of dogs tested on the pointing gesture. It has also been observed that when actively approaching a target (as required here), dogs’ spontaneous preferences may override other available information. For instance, in the study of Kubinyi et al. (2020), dogs’ gazing direction between two objects was aligned with their owner's expressed preference, but fetching was dominated by their own preference.

#### Sleep spindles and relative latency reduction

Pearson's correlations were used to compare relative latency reduction with fast spindle density during TMR but also with averaged (within subjects) fast spindle density pooled from across both adaptation and experimental recordings. Averaged density was recently demonstrated in dogs to display a stronger relationship with learning performance (Iotchev et al., 2020a) which is thought to derive from reducing measurement error while approximating interindividual trait density.

**Figure 6.**
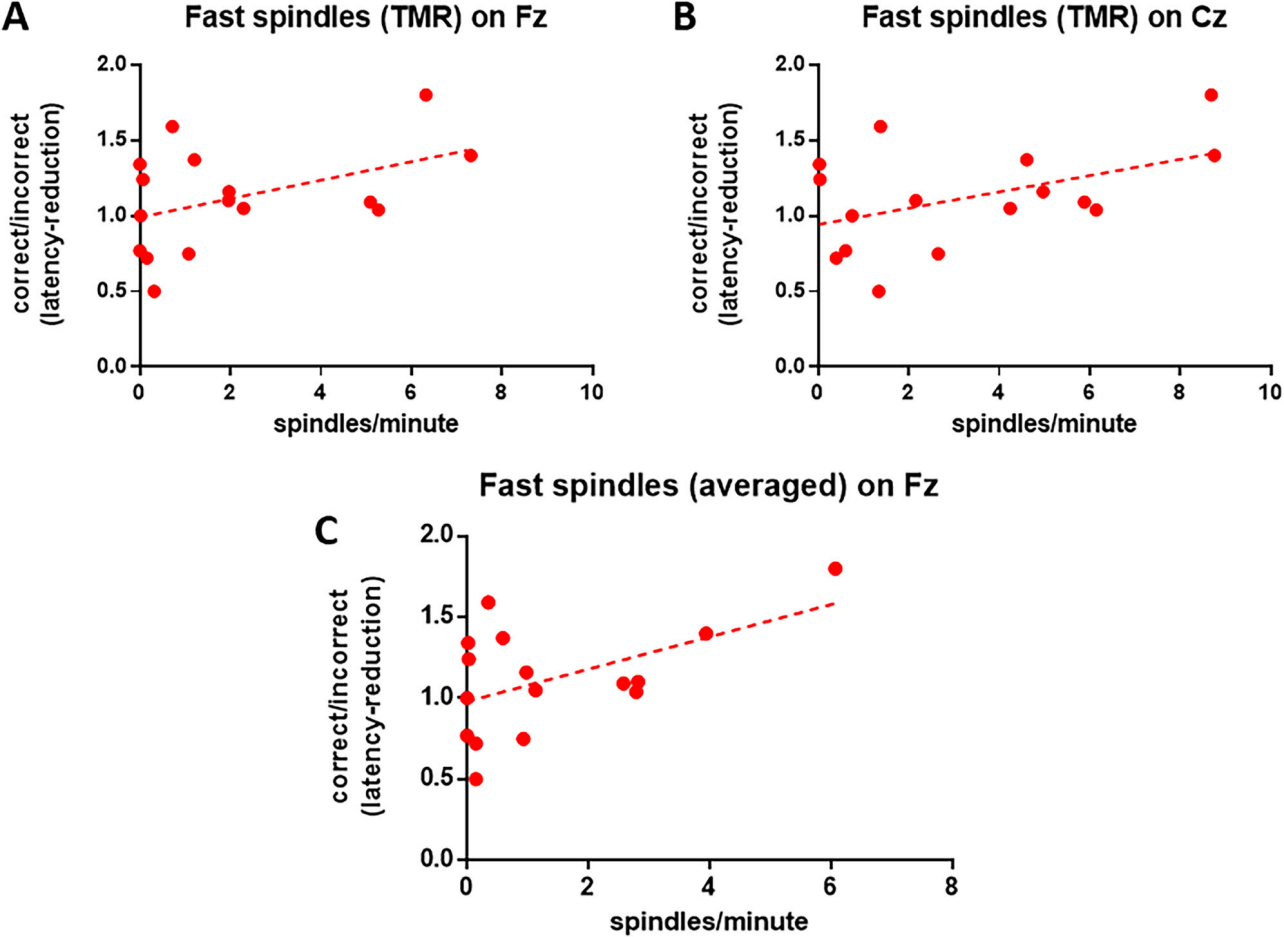
Relative latency reduction (correct/incorrect trials) and fast (>13 Hz) density on Fz (***A***) and Cz (***B***) and averaged across both TMR and adaptation recordings on Fz (***C***).

Averaged fast spindle density on Fz was significantly correlated with relative latency reduction (*r* = 0.514; *p* = 0.042), but not on Cz (*p* = 0.128). There was also a trend for a positive association between relative latency reduction and fast spindle density during TMR on Fz (*p* = 0.085) and Cz (*p* = 0.068). These associations are plotted in Figure 6.

## Discussion

The present study is a pioneering effort in adapting a fully noninvasive TMR paradigm in general and in investigating the TMR effect in a visuospatial learning task in particular on family dogs. First of all, our results provide evidence that TMR is applicable to dogs, as the macrostructural variables were similar to those in previous studies applying sleep polysomnography (Kis et al., 2014), indicating that the TMR intervention did not significantly influence dogs’ natural sleeping patterns. Therefore, we conclude that although the TMR procedure was feasible, we did not find strong evidence for a TMR effect on learning. We assume that the learning task was too difficult for the dogs, with the three nonwords resulting in relatively low accuracy scores and a high dropout rate. Analyses that were conducted in response to the initial results suggest that a different measure of learning could be more sensitive by emphasizing response latency rather than response accuracy. This will be discussed in more detail after looking at the main findings first.

Our results yielded a convincing demonstration of a physiological TMR effect, although a cueing benefit on learning was not reflected in choice accuracy. Specifically, we observed a higher density (occurrence/minute) of fast sleep spindles during TMR recordings, compared with two reference populations: adaptation recordings from the same dogs and a larger collection of adaptation recordings from a previous study. We chose to compare TMR and non-TMR recordings for analyzing possible effects on sleep spindles due to the prevailing observation in the literature that TMR leads to increases in (fast) spindle density (Cairney et al., 2018; Carbone et al., 2021; Hopper, 2021). Since our paradigm was not set up for comparing TMR with a control condition, non-TMR recordings from a larger pool of data were added to our analysis to have a more reliable estimate of “normal” sleep spindle occurrence and hence determine TMR-induced increases with more certainty. All recordings were capturing daytime naps, and although TMR recordings were longer, sleep spindle occurrence was measured per minute to account for differences in non-REM sleep duration between individuals and conditions. Moreover, sleep spindle density in some of our previous datasets was negatively correlated with non-REM duration (Iotchev et al., 2017); thus, the length of TMR recordings was not deemed a likely alternative explanation for the observation. Having included a large reference population of non-TMR recordings, we could demonstrate increases in fast sleep spindle density during TMR that were far above the norm. This did not extend to slow spindles. A higher occurrence of specifically fast sleep spindles during TMR was also observed by Cairney et al. (2018) and by Carbone et al. (2021). However, the topography and speed of spindles involved in learning may depend on the type of learning task (Clemens et al., 2005, 2006), cortical areas most involved during acquisition (Petzka et al., 2022), or additional drug interventions (Carbone et al., 2021) more than on TMR. TMR-induced increases in sleep spindles without a clear cueing benefit were also observed by Hopper (2021), but an association with learning performance is more commonly reported (Creery et al., 2015; Cairney et al., 2018; Wang et al., 2019; Carbone et al., 2021).

Our findings showed that dogs had shorter choice latencies after sleep compared with the baseline, indicating a potential effect of sleep on task performance irrespective of the handling. This aligns with previous studies that have demonstrated the role of sleep in memory consolidation in dogs, particularly in tasks which involve learning in a social context (Kis et al., 2017). The observed reduction in latency suggests that sleep may facilitate recall or decision-making, consistent with the understanding that sleep enhances cognitive function, as has been widely reported in both human and nonhuman animal studies (Diekelmann and Born, 2010; Barnes and Wilson, 2014). However, unlike the strong associations seen in human studies between sleep stages, such as REM, and memory consolidation, the effect here was modest, which may be due to either the different nature of the task or the TMR protocol used. The observation of an overall reduced response latency also prompted us to further explore latency reduction as an alternative for quantifying learning success. The majority of dogs displayed a stronger latency reduction for correct trials, but we did not observe a statistically significant difference between correct and incorrect trials regarding latency reduction.

The extremely high fast spindle densities observed in TMR recordings and the postsleep reduction in response latency led us to explore a few post hoc analyses, which may indicate a cueing benefit other than the one we expected to observe in the animals’ choice behavior. For this we devised a measure of relative latency reduction which expresses how much stronger latency was reduced postsleep for correct trials than for incorrect trials (a further distinction into cued and uncued trials was not feasible from a stand point of sample sizes and statistical power). This relative latency reduction displayed a positive association with fast spindle density; however, this effect was only significant when considering averaged density (across both available recordings of the participating dogs), whereas considering only TMR recordings this association was a trend. This is the second time that we observe averaged sleep spindle density to be a better predictor of cognitive performance in dogs (Iotchev et al., 2020a). This may be due to reduced measurement error and thus a cleaner estimate of the underlying trait density. In humans, sleep spindle occurrence tends to be stable across recordings (Silverstein and Levy, 1976), and this is a heritable trait (Goldschmied et al., 2021). It has been argued, however, that several recording instances are needed to reliably estimate some spindle traits, e.g., slow spindle density in human adolescents (Reynolds et al., 2019). Our previous and current findings in the dog on the predictive power of averaged, across recordings, spindle density likewise support this proposition. The importance of trait and state density as predictors of cognition, meanwhile, are not mutually exclusive. Although there are stronger interindividual than intraindividual differences in sleep spindle density, in both humans and dogs, density additionally increases after engagement with novel tasks (Gais et al., 2002; Iotchev et al., 2017). Therefore, the increase in fast spindle density observed for TMR recordings paired with fast spindle density being linked to stronger latency reduction for correct trials suggests that TMR in this study might have exhibited a cueing benefit to some extent. Importantly, this is the first demonstration of fast spindles being linked to learning in the dog, where so far slow spindle density has been the predominant correlate of postsleep recall (Iotchev et al., 2017, 2020a). This may reflect task-specific learning demands. In the present work, fast spindle density correlates with a measure of learning derived from latency reduction, likewise Tamaki et al. (2008) found latency reduction in human subjects on a visuomotor task to be associated with fast sleep spindles. Generally, in the human literature, fast sleep spindles are more commonly associated with motor learning (Morin et al., 2008; Tamaki et al., 2008, 2009; Lustenberger et al., 2016), whereas slow spindles appear to support word–pair associations instead (Clemens et al., 2005; Schmidt et al., 2006; Lustenberger et al., 2015; Kuula et al., 2019).

The hypothesized higher cueing benefit for previously more successful dogs was not supported by the data. This is contradictory to human findings where presleep performance seemed to be a significant predictor of cueing effectiveness (Creery et al., 2015). One possible explanation for this discrepancy could be species differences in memory processing or the specific type of memory task employed. While, in humans, stronger presleep performance often leads to more robust memory reactivation during sleep, the same mechanism might not apply as strongly in dogs, or the TMR cues used may not have been sufficiently salient to enhance performance selectively in dogs with higher presleep accuracy. Future research should test the effect with odor cues, because olfaction is a more prominent domain in perception in case of dogs (Kokocińska-Kusiak et al., 2021); therefore the effect of cueing benefit or TMR may be more easily detected.

Furthermore, another result of our study was the effect of learning on sleep parameters. Dogs who performed better in the presleep baseline task exhibited increased EEG activity, particularly in the alpha range during drowsiness, similar to what was observed in human studies (Rihm et al., 2014; Schreiner et al., 2015). This suggests that, like in humans, certain EEG markers during sleep may be indicative of learning and memory processes in dogs. The identification of a significant Rüger area in the alpha range supports this, although the functional significance of these findings in dogs requires further investigation. The absence of significant changes in other sleep parameters, such as delta power, suggests that the relationship between sleep architecture and memory reactivation might differ between species or depend on the specific memory task.

In comparison with rodent models where TMR often involves fear conditioning paradigms (Hars and Hennevin, 1987; Barnes and Wilson, 2014), the use of a visuospatial task in dogs presents a closer parallel to human studies. The limited evidence for TMR's effectiveness in enhancing memory recall in dogs, however, suggests that further refinement of the paradigm may be necessary to achieve results comparable with those seen in humans. For example, the present study contained only 15 trials (5 trials per hiding place) which is a relatively low number compared with human studies, where there are usually ∼60 trials per condition with 60 different words (Cairney et al., 2014). Evidently, an average dog cannot learn 60 words in a single test situation; therefore, this limitation was inevitable. Future research should aim to recruit from a specific subgroup of dogs, e.g., the “gifted word learners,” who can memorize up to 12 words in a single learning session (Dror et al., 2021).

The results of this study, while not entirely supportive of the initial hypotheses, provide valuable groundwork for further investigation of the possible TMR effect in dogs. As our study was the first attempt in this field and used nonword commands, TMR can be tested with varying cues (e.g., to odor cues), or it is possible to extend the training period to enhance cueing benefit. Possibly choosing another easier and more familiar type of learning (like in Kis et al., 2017) could also bring new advantages to the field. Additionally, exploring different types of memory tasks and their interaction with sleep stages could yield further insights into the mechanisms underlying memory consolidation in dogs.

## Synthesis

Reviewing Editor: Laura Bradfield, University of Technology Sydney Decisions are customarily a result of the Reviewing Editor and the peer reviewers coming together and discussing their recommendations until a consensus is reached. When revisions are invited, a fact-based synthesis statement explaining their decision and outlining what is needed to prepare a revision will be listed below. The following reviewer(s) agreed to reveal their identity: NONE.

Thank you for your re-submission of the registered report. I found the manuscript to be very interesting. I just have some minor edits to be implemented. Also can you please include a significance statement?

According to the eNeuro website, this is a 120 word (approx.) statement "The Significance Statement should provide a clear explanation of the importance and relevance of the research in a manner accessible to researchers without specialist knowledge in the field and informed lay readers. The Significance Statement will appear within the paper below the abstract."

Please also implement the following changes:

• TMR - acronym not spelled out the first time in abstract

• Not sure what the authors mean by 'family' dogs? Is this to distinguish them from laboratory animals? Please could this be made clearer.

• Line 64 - a full stop (period) needs to be inserted after "barely explored".

• Line 93, 'a' should not be capitalised

• Lines 132-133 do the authors know information about the specific breeds used? If so can they please include it?

• Line 129, could the authors please define "center bias"

• Line 202 - do you mean 'site' or 'side'?

• Line 244 - should read "two other ones" not "other two ones"

• Line 260 - suggest changing "E" to "experimenter", and anywhere else this might apply (e.g. Line 269).

• Line 355 "All GLMM models will be conducted with and without dogs who showed side preference in the baseline trials (i.e. 9/15 choices)" - this is crossed out. Why is that? Was this analysis not carried out, and if not, why not?

• Line 364 - why did you not test the correlations with all macrostructural variables and bin-by-bin analysis if you wanted to? I think you need to state why in the manuscript.

• Line 404 - Therefor - needs an e on the end

• Line 432 - wasobserved needs a space

## References

[B1] Abdai J, Miklósi Á (2015) Family dog project©: history and future of the ethological approach to human-dog interaction. Zeszyty Naukowe Uniwersytetu Przyrodniczego We Wroclawiu: Biologia i Hodowla Zwierzat 79(613).

[B2] Abt K (1987) Descriptive data analysis: a concept between confirmatory and exploratory data analysis. Methods Inf Med 26:77–88. 10.1055/s-0038-1635488 3587055

[B3] Antony JW, Piloto L, Wang M, Pacheco P, Norman KA, Paller KA (2018) Sleep spindle refractoriness segregates periods of memory reactivation. Curr Biol 28:1736–1743. 10.1016/j.cub.2018.04.020 29804809 PMC5992601

[B4] Bailey J, Pereira S (2018) Advances in neuroscience imply that harmful experiments in dogs are unethical. J Med Ethics 44:47–52. 10.1136/medethics-2016-103630 28739639 PMC5749309

[B5] Barnes DC, Wilson DA (2014) Slow-wave sleep-imposed replay modulates both strength and precision of memory. J Neurosci 34:5134–5142. 10.1523/JNEUROSCI.5274-13.2014 24719093 PMC3983797

[B6] Bódizs R, Kis A, Gácsi M, Topál J (2020) Sleep in the dog: comparative, behavioral and translational relevance. Curr Opin Behav Sci 33:25–33. 10.1016/j.cobeha.2019.12.006

[B7] Bolló H, File B, Tópal J, Kis A (2023) Side bias behaviour in dogs shows parallels to the hemispatial neglect syndrome. Appl Anim Behav Sci 263:105921. 10.1016/j.applanim.2023.105921

[B8] Bolló H, Kovács K, Lefter R, Gombos F, Kubinyi E, Topál J, Kis A (2020) REM versus non-REM sleep disturbance specifically affects inter-specific emotion processing in family dogs (*Canis familiaris*). Sci Rep 10:10492. 10.1038/s41598-020-67092-5 32591578 PMC7319983

[B9] Born J, Rasch B, Gais S (2006) Sleep to remember. Neuroscientist 12:410–424. 10.1177/1073858406292647 16957003

[B10] Bunford N, Andics A, Kis A, Miklósi Á, Gácsi M (2017) *Canis familiaris* as a model for non-invasive comparative neuroscience. Trends Neurosci 40:438–452. 10.1016/j.tins.2017.05.003 28571614

[B11] Cairney SA, Durrant SJ, Hulleman J, Lewis PA (2014) Targeted memory reactivation during slow wave sleep facilitates emotional memory consolidation. Sleep 37:701–707. 10.5665/sleep.3572 24688163 PMC3954173

[B12] Cairney SA, Guttesen AV, Marj NE, Staresina BP (2018) Memory consolidation is linked to spindle-mediated information processing during sleep. Curr Biol 28:948–954. 10.1016/j.cub.2018.01.087 29526594 PMC5863764

[B13] Carbone J, Bibián C, Reischl P, Born J, Forcato C, Diekelmann S (2021) The effect of zolpidem on targeted memory reactivation during sleep. Learn Mem 28:307–318. 10.1101/lm.052787.120 34400532 PMC8372567

[B14] Clemens Z, Fabó D, Halász P (2005) Overnight verbal memory retention correlates with the number of sleep spindles. Neuroscience 132:529–535. 10.1016/j.neuroscience.2005.01.011 15802203

[B15] Clemens Z, Fabó D, Halász P (2006) Twenty-four hours retention of visuospatial memory correlates with the number of parietal sleep spindles. Neurosci Lett 403:52–56. 10.1016/j.neulet.2006.04.035 16714084

[B16] Cox R, Hofman WF, de Boer M, Talamini LM (2014) Local sleep spindle modulations in relation to specific memory cues. Neuroimage 99:103–110. 10.1016/j.neuroimage.2014.05.028 24852461

[B17] Creery JD, Oudiette D, Antony JW, Paller KA (2015) Targeted memory reactivation during sleep depends on prior learning. Sleep 38:755–763. 10.5665/sleep.4670 25515103 PMC4402655

[B18] Diekelmann S, Biggel S, Rasch B, Born J (2012) Offline consolidation of memory varies with time in slow wave sleep and can be accelerated by cuing memory reactivations. Neurobiol Learn Mem 98:103–111. 10.1016/j.nlm.2012.07.002 22789831

[B19] Diekelmann S, Born J (2010) The memory function of sleep. Nat Rev Neurosci 11:114. 10.1038/nrn2762 20046194

[B20] Dror I, Melinek J, Arden JL, Kukucka J, Hawkins S, Carter J, Atherton DS (2021) Cognitive bias in forensic pathology decisions. J Forensic Sci 66:1751–1757. 10.1111/1556-4029.14697 33608908 PMC8451910

[B21] Druzhkova AS, Thalmann O, Trifonov VA, Leonard JA, Vorobieva NV, Ovodov ND, Graphodatsky AS, Wayne RK (2013) Ancient DNA analysis affirms the canid from Altai as a primitive dog. PLoS One 8:e57754. 10.1371/journal.pone.0057754 23483925 PMC3590291

[B22] Gácsi M, Kara E, Belényi B, Topál J, Miklósi Á (2009) The effect of development and individual differences in pointing comprehension of dogs. Anim Cogn 12:471–479. 10.1007/s10071-008-0208-6 19130102

[B23] Gais S, Mölle M, Helms K, Born J (2002) Learning-dependent increases in sleep spindle density. J Neurosci 22:6830–6834. 10.1523/JNEUROSCI.22-15-06830.2002 12151563 PMC6758170

[B24] Genzel L, Kroes MC, Dresler M, Battaglia FP (2014) Light sleep versus slow wave sleep in memory consolidation: a question of global versus local processes? Trends Neurosci 37:10–19. 10.1016/j.tins.2013.10.002 24210928

[B25] Gergely A, Topál J, Dóka A, Miklósi Á (2014) Dogs are able to generalise directional acoustic signals to different contexts and tasks. Appl Anim Behav Sci 156:54–61. 10.1016/j.applanim.2014.04.005

[B26] Goldschmied JR, et al. (2021) Spindles are highly heritable as identified by different spindle detectors. Sleep 44:zsaa230. 10.1093/sleep/zsaa230 33165618 PMC8033448

[B27] Hare B, Tomasello M (2005) Human-like social skills in dogs? Trends Cogn Sci 9:439–444. 10.1016/j.tics.2005.07.003 16061417

[B28] Hars B, Hennevin E (1987) Impairment of learning by cueing during postlearning slow-wave sleep in rats. Neurosci Lett 79:290–294. 10.1016/0304-3940(87)90446-0 3658221

[B29] Hopper JW (2021) Investigating the role of targeted memory reactivation in sleep spindle production. The University of Western Ontario, Canada.

[B30] Iotchev IB, Kis A, Bódizs R, Van Luijtelaar G, Kubinyi E (2017) EEG transients in the sigma range during non-REM sleep predict learning in dogs. Sci Rep 7:12936. 10.1038/s41598-017-13278-3 29021536 PMC5636833

[B31] Iotchev IB, Kis A, Turcsán B, Tejeda Fernández de Lara DR, Reicher V, Kubinyi E (2019) Age-related differences and sexual dimorphism in canine sleep spindles. Sci Rep 9:10092. 10.1038/s41598-019-46434-y 31300672 PMC6626048

[B32] Iotchev IB, Kubinyi E (2021) Shared and unique features of mammalian sleep spindles-insights from new and old animal models. Biol Rev 96:1021–1034. 10.1111/brv.12688 33533183

[B33] Iotchev IB, Reicher V, Kovács E, Kovács T, Kis A, Gácsi M, Kubinyi E (2020a) Averaging sleep spindle occurrence in dogs predicts learning performance better than single measures. Sci Rep 10:22461. 10.1038/s41598-020-80417-8 33384457 PMC7775433

[B34] Iotchev IB, Szabó D, Kis A, Kubinyi E (2020b) Possible association between spindle frequency and reversal-learning in aged family dogs. Sci Rep 10:6505. 10.1038/s41598-020-63573-9 32300165 PMC7162895

[B35] Iotchev IB, Szabó D, Turcsán B, Bognár Z, Kubinyi E (2024) Sleep-spindles as a marker of attention and intelligence in dogs. Neuroimage 303:120916. 10.1016/j.neuroimage.2024.120916 39505225

[B36] Kaminski J, Call J, Fischer J (2004) Word learning in a domestic dog: evidence for “fast mapping”. Science 304:1682–1683. 10.1126/science.1097859 15192233

[B37] Kis A, Szakadát S, Gácsi M, Kovács E, Simor P, Török C, Gombos F, Bódizs R, Topál J (2017) The interrelated effect of sleep and learning in dogs (*Canis familiaris*); an EEG and behavioural study. Sci Rep 7:41873. 10.1038/srep41873 28165489 PMC5292958

[B38] Kis A, Szakadát S, Kovács E, Gácsi M, Simor P, Gombos F, Topál J, Miklósi Á, Bódizs R (2014) Development of a non-invasive polysomnography technique for dogs (*Canis familiaris*). Physiol Behav 130:149–156. 10.1016/j.physbeh.2014.04.004 24726397

[B39] Kokocinska-Kusiak A, Woszczylo M, Zybala M, Maciocha J, Barlowska K, Dzieciol M (2021) Canine olfaction: physiology, behavior, and possibilities for practical applications. Animals 11:2463. 10.3390/ani11082463 34438920 PMC8388720

[B40] Kubinyi E, Szánthó F, Gilmert E, Iotchev IB, Miklósi Á (2020) Human expressions of object preference affect dogs’ perceptual focus, but not their action choices. Front Psychol 11:588916. 10.3389/fpsyg.2020.588916 33240181 PMC7677580

[B41] Kuula L, Tamminen J, Makkonen T, Merikanto I, Räikkönen K, Pesonen AK (2019) Higher sleep spindle activity is associated with fewer false memories in adolescent girls. Neurobiol Learn Mem 157:96–105. 10.1016/j.nlm.2018.12.005 30553019

[B42] Latchoumane CFV, Ngo HVV, Born J, Shin HS (2017) Thalamic spindles promote memory formation during sleep through triple phase-locking of cortical, thalamic, and hippocampal rhythms. Neuron 95:424–435. 10.1016/j.neuron.2017.06.025 28689981

[B43] Lustenberger C, Boyle MR, Alagapan S, Mellin JM, Vaughn BV, Fröhlich F (2016) Feedback-controlled transcranial alternating current stimulation reveals a functional role of sleep spindles in motor memory consolidation. Curr Biol 26:2127–2136. 10.1016/j.cub.2016.06.044 27476602 PMC4996680

[B44] Lustenberger C, Wehrle F, Tüshaus L, Achermann P, Huber R (2015) The multidimensional aspects of sleep spindles and their relationship to word-pair memory consolidation. Sleep 38:1093–1103. 10.5665/sleep.4820 25845686 PMC4481015

[B45] Mölle M, Eschenko O, Gais S, Sara SJ, Born J (2009) The influence of learning on sleep slow oscillations and associated spindles and ripples in humans and rats. Eur J Neurosci 29:1071–1081. 10.1111/j.1460-9568.2009.06654.x 19245368

[B46] Morin A, Doyon J, Dostie V, Barakat M, Tahar AH, Korman M, Benali H, Kami A, Ungerleider LG, Carrier J (2008) Motor sequence learning increases sleep spindles and fast frequencies in post-training sleep. Sleep 31:1149–1156. 18714787 PMC2542961

[B47] Nonclercq A, Urbain C, Verheulpen D, Decaestecker C, Van Bogaert P, Peigneux P (2013) Sleep spindle detection through amplitude-frequency normal modelling. J Neurosci Methods 214:192–203. 10.1016/j.jneumeth.2013.01.015 23370313

[B48] Oudiette D, Antony JW, Creery JD, Paller KA (2013) The role of memory reactivation during wakefulness and sleep in determining which memories endure. J Neurosci 33:6672–6678. 10.1523/JNEUROSCI.5497-12.2013 23575863 PMC3677604

[B49] Parker HG, Shearin AL, Ostrander EA (2010) Man’s best friend becomes biology’s best in show: genome analyses in the domestic dog. Annu Rev Genet 44:309–336. 10.1146/annurev-genet-102808-115200 21047261 PMC3322674

[B50] Petzka M, Chatburn A, Charest I, Balanos GM, Staresina BP (2022) Sleep spindles track cortical learning patterns for memory consolidation. Curr Biol 32:2349–2356. 10.1016/j.cub.2022.04.045 35561681 PMC9616732

[B51] Range F, Hentrup M, Virányi Z (2011) Dogs are able to solve a means-end task. Anim Cogn 14:575–583. 10.1007/s10071-011-0394-5 21445577 PMC3947724

[B52] Rasch B, Born J (2013) About sleep’s role in memory. Physiol Rev 93:681–766. 10.1152/physrev.00032.2012 23589831 PMC3768102

[B53] Reicher V, Kis A, Simor P, Bódizs R, Gombos F, Gácsi M (2020) Repeated afternoon sleep recordings indicate first-night-effect-like adaptation process in family dogs. J Sleep Res e12998. 10.1111/jsr.12998 32067296

[B54] Reynolds CM, Gradisar M, Short MA (2019) Reliability of sleep spindle measurements in adolescents: how many nights are necessary? J Sleep Res 28:e12698. 10.1111/jsr.12698 29736916

[B55] Rihm JS, Diekelmann S, Born J, Rasch B (2014) Reactivating memories during sleep by odors: odor specificity and associated changes in sleep oscillations. J Cogn Neurosci 26:1806–1818. 10.1162/jocn_a_00579 24456392

[B56] Rudoy JD, Voss JL, Westerberg CE, Paller KA (2009) Strengthening individual memories by reactivating them during sleep. Science 326:1079. 10.1126/science.1179013 19965421 PMC2990343

[B57] Schmidt C, Peigneux P, Muto V, Schenkel M, Knoblauch V, Münch M, De Quervain DJ, Wirz-Justice A, Cajochen C (2006) Encoding difficulty promotes postlearning changes in sleep spindle activity during napping. J Neurosci 26:8976–8982. 10.1523/JNEUROSCI.2464-06.2006 16943553 PMC6675334

[B58] Schouten DI, Pereira SI, Tops M, Louzada FM (2017) State of the art on targeted memory reactivation: sleep your way to enhanced cognition. Sleep Med Rev 32:123–131. 10.1016/j.smrv.2016.04.002 27296303

[B59] Schreiner T, Lehmann M, Rasch B (2015) Auditory feedback blocks memory benefits of cueing during sleep. Nat Commun 6:8729. 10.1038/ncomms9729 26507814 PMC4640077

[B60] Silverstein LD, Levy CM (1976) The stability of the sigma sleep spindle. Electroencephalogr Clin Neurophysiol 40:666–670. 10.1016/0013-4694(76)90142-5 57053

[B61] Takahashi Y, Ebihara S, Nakamura Y, Nishi C, Takahashi K (1972) Circadian sleep and waking patterns in the laboratory dog. Sleep Res 1:144.

[B62] Tamaki M, Matsuoka T, Nittono H, Hori T (2008) Fast sleep spindle (13–15 Hz) activity correlates with sleep-dependent improvement in visuomotor performance. Sleep 31:204–211. 10.1093/sleep/31.2.204 18274267 PMC2225572

[B63] Tamaki M, Matsuoka T, Nittono H, Hori T (2009) Activation of fast sleep spindles at the premotor cortex and parietal areas contributes to motor learning: a study using SLORETA. Clin Neurophysiol 120:878–886. 10.1016/j.clinph.2009.03.006 19376746

[B64] Topál J, Miklósi Á, Gácsi M, Dóka A, Pongrácz P, Kubinyi E, Virányi Z, Csányi V (2009) The dog as a model for understanding human social behavior. In: *Advances in the study of behavior* (Brockmann HJ, Roper TJ, Naguib M, Wynne-Edwards KE, Mitani JC, Simmons LW, eds), Vol. 39, pp 71–116. Burlington: Academic Press.

[B65] Vorster AP, Born J (2015) Sleep and memory in mammals, birds and invertebrates. Neurosci Biobehav Rev 50:103–119. 10.1016/j.neubiorev.2014.09.020 25305058

[B66] Wang B, Antony JW, Lurie S, Brooks PP, Paller KA, Norman KA (2019) Targeted memory reactivation during sleep elicits neural signals related to learning content. J Neurosci 39:6728–6736. 10.1523/JNEUROSCI.2798-18.2019 31235649 PMC6703880

[B67] Zepelin H (1994) Mammalian sleep. In: *Principles and practice of sleep medicine* (Kryger MH, Roth T, Dement WC, eds), pp 69–80. Philadelphia: W. B. Saunders.

